# Analysis of Risk Factor Changes for Myopia in Korean Adolescents Before and After the COVID-19 Pandemic

**DOI:** 10.3390/medicina61101798

**Published:** 2025-10-06

**Authors:** Seeun Lee, So Ra Kim, Mijung Park

**Affiliations:** 1 Department of Optometry, Seoyeong University, Paju 10843, Republic of Korea; 2 Department of Optometry, Seoul National University of Science and Technology, Seoul 01811, Republic of Korea

**Keywords:** myopia, COVID-19, adolescent, refractive errors, BMI, near work time

## Abstract

*Background and Objectives*: To analyze changes in risk factors for refractive errors among Korean adolescents before and after the COVID-19 pandemic and examine the impact of lifestyle modifications on myopia development, *Methods*: this cross-sectional study utilized nationally representative data from the Korea National Health and Nutrition Examination Survey (KNHANES) VII (2016) and VIII (2021). We analyzed 691 adolescents aged 10–18 years from 2016 (pre-COVID-19) and 490 from 2021 (post-COVID-19). Refractive errors were categorized as hyperopia (≥+0.50 D), emmetropia (−0.50 to +0.50 D), myopia (−6.00 to −0.50 D), and high myopia (≤−6.00 D). Complex sample linear regression analyses identified factors associated with spherical equivalent (SE) refractive errors. *Results:* At the population level, overall myopia prevalence declined from 84.2% in 2016 to 77.4% in 2021, whereas the prevalence of high myopia increased from 10.0% to 11.5% (*p* = 0.047). This indicates that although the absolute proportion of adolescents with myopia decreased, the relative contribution of high myopia to the overall myopia burden within this population increased. Mean SE was −2.77 ± 0.11 D in (−10.63~+3.00 D/median: −2.00 D) 2016 and −2.63 ± 0.13 D (−14.00~+1.63/median: −1.75 D) in 2021 (*p* = 0.443). Age-related myopia progression accelerated post-pandemic (−0.193 D to −0.324 D per year in univariate regression and −0.185 D to −0.312 D, in multivariate regression analysis, *p* < 0.001). In both the 3 h and ≥4 h near work groups, statistically significant associations were observed at both time points, but a greater myopic shift was evident after COVID-19 (B = –0.853 and –0.757 in 2016; B = –1.311 and –1.167 in 2021, *p* < 0.05). *Conclusions:* The COVID-19 pandemic altered myopia risk factors among Korean adolescents. High myopia prevalence increased despite overall myopia with underweight status and environmental factors such as digital device time and urban living identified as important considerations for post-pandemic myopia prevention.

## 1. Introduction

Myopia is a common refractive error in which distant objects appear blurred because images are focused in front of the retina, and its prevalence is predicted to increase worldwide.1 Region al prevalence studies have consistently shown that myopia prevalence is significantly higher in East and Southeast Asia compared to North America, Europe, and Africa [1,2]. Numerous studies on the prevalence of myopia among Korean adolescents have reported a prevalence ranging from 50% to 90% [3,4,5].

The development and progression of refractive errors, including myopia, are influenced by a complex interaction of genetic and environmental factors. When parents have myopia, the risk of myopia development in their children increases significantly [6,7], and twin studies have confirmed that genetic factors play an important role in refractive errors [8,9]. However, myopia cannot be explained solely by genetic factors, and environmental factors such as increased near work, decreased outdoor activities, and body mass index are known to play crucial roles [10,11,12].

The COVID-19 pandemic that began in 2020 caused drastic changes in lifestyle worldwide., particularly affecting the lifestyle of adolescents during the critical period when refractive errors increase [13,14,15]. ^15^Due to social distancing measures, remote learning was greatly expanded in Korea, and as outdoor activities were restricted, the use of display devices such as smartphones, computers, and tablet PCs increased dramatically while physical activity time decreased substantially. Additionally, decreased physical activity and increased obesity rates among adolescents may act as additional risk factors for myopia [16,17]. In addition to traditional forms of near work such as reading and writing, digital device use has increasingly been recognized as a distinct risk factor for myopia [10]. The COVID-19 pandemic markedly accelerated exposure to digital screens, underscoring the necessity of evaluating its potential impact on refractive error development. While extensive research has been conducted on major risk factors for refractive errors, studies comparing and analyzing refractive error risk factors before and after the COVID-19 pandemic remain limited. This study hypothesizes that the COVID-19 pandemic heightened the influence of myopia risk factors in adolescents and examines the impact of related physical and lifestyle changes on refractive errors. Through this, we aim to provide foundational data for refractive error prevention and management strategies helping to contribute to future public health policy development.

## 2. Materials and Methods

### 2.1. Study Design and Setting

This cross-sectional study utilized data from the KNHANES, a nationwide, population-based health survey conducted by the Korea Disease Control and Prevention Agency and the Ministry of Health and Welfare. We compared data from KNHANES VII (2016) representing the pre-COVID-19 period and KNHANES VIII (2021) representing the post-COVID-19 period to examine changes in refractive error risk factors among Korean adolescents.

### 2.2. Participants

Eligibility criteria and participant selection

The target population comprised Korean adolescents aged 10–18 years who participated in ophthalmologic examinations during KNHANES VII (2016) and KNHANES VIII (2021).

**Figure 1:**
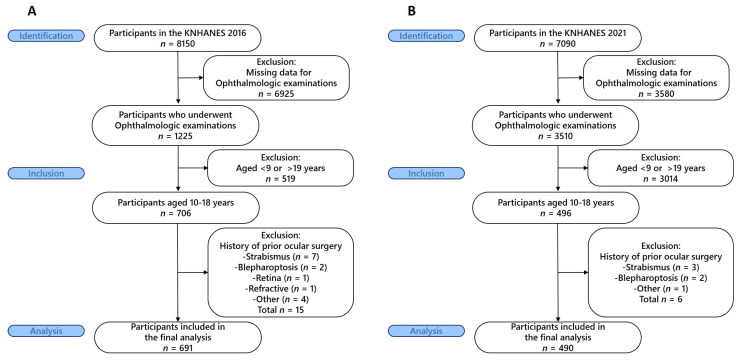
STROBE flow diagram of participant selection from the 2016 (**A**) and 2021 (**B**) KNHANES.

For the 2016 survey (pre-COVID-19), among 8150 total KNHANES participants, 1225 underwent ophthalmologic examinations. Of these, 706 were adolescents aged 10–18 years. We excluded participants with a history of ocular surgery including strabismus (*n* = 7), blepharoptosis (*n* = 2), retinal surgery (*n* = 1), refractive surgery (*n* = 1), and other ocular surgeries (*n* = 4), resulting in 691 participants for final analysis (Figure 1A).

For the 2021 survey (post-COVID-19), among 7090 total KNHANES participants, 3510 underwent ophthalmologic examinations. Of these, 496 were adolescents aged 10–18 years. We excluded participants with a history of ocular surgery including strabismus (*n* = 3), blepharoptosis (*n* = 2), and other ocular surgeries (*n* = 1), resulting in 490 participants for final analysis (Figure 1B). The KNHANES were conducted according to the Declaration of Helsinki and approved by the Institutional Review Board of the Korea Centers for Disease Control and Prevention. All participants provided written informed consent.

### 2.3. Variables

#### 2.3.1. Outcome Measures

The primary outcome was refractive error status, measured as spherical equivalent (SE) refractive error. Refractive errors were assessed using an autorefractor (KR8800, Topcon, Tokyo, Japan) without cycloplegia. SE was calculated as sphere + 1/2 cylinder power and categorized into four groups: hyperopia (SE ≥ +0.50 diopters (D)), emmetropia (−0.50 < SE < +0.50 D), myopia (−6.00 < SE ≤ −0.50 D), and high myopia (SE ≤ −6.00 D). The right eye measurements were used for analysis as there was no significant difference between both eyes (*p* > 0.05).

#### 2.3.2. Exposure Variables

Demographic variables included age, sex, and residential area (urban/rural). Socioeconomic variables included equivalized household income categorized into quartiles. Anthropometric measurements included body mass index (BMI), calculated as weight (kg) divided by height (m)^2^ and categorized according to the 2017 Korean Children and Adolescents Growth Charts: underweight (<5th percentile), normal weight (5th to <85th percentile), overweight (85th to <95th percentile), and obese (≥95th percentile).

Lifestyle factors included sleep duration, stress perception, sedentary time, and near work activities. Sleep duration was calculated as the weighted average of weekday and weekend sleep hours (5 weekdays + 2 weekend days)/7. Stress perception was assessed using a 4-point scale, with participants reporting “very much” or “much” stress classified as having high perceived stress.

Near work time assessment differed between survey years due to questionnaire modifications. In 2016, participants were asked about total daily near work time including both reading and computer work “During the past year, on average, how many hours per day did you spend on near work activities (e.g., computer use, reading books)?”. In 2021, reading time (books, newspapers, documents) and digital device usage time (smartphones, tablets, computers) were assessed separately: “During the past year, on average, how many hours per day did you spend reading printed materials (books, newspapers, documents)?”/“During the past year, on average, how many hours per day did you spend using digital devices (smartphones, tablets, computers)?”.

#### 2.3.3. Statistical Methods

All statistical analyses were performed using SPSS Statistics version 23.0, incorporating complex sample analysis procedures to account for the stratified, clustered sampling design and sampling weights of KNHANES.

Descriptive statistics were calculated for all variables, with categorical variables presented as weighted percentages and continuous variables as weighted means with standard errors. -Rao-Scott chi-square tests were used to compare categorical variables between refractive error groups, while complex sample general linear models were used for continuous variables. Corrected F statistic from the Rao–Scott chi-square test was reported as a measure of within-group variability. To identify factors associated with refractive errors, we performed both univariate and multivariate complex sample linear regression analyses using SE as a continuous outcome variable. Results are presented as regression coefficients with 95% confidence intervals. Statistical significance was set at *p* < 0.05.

## 3. Results

### 3.1. Changes in Refractive Error Distribution Before and After COVID-19

**Figure 2:**
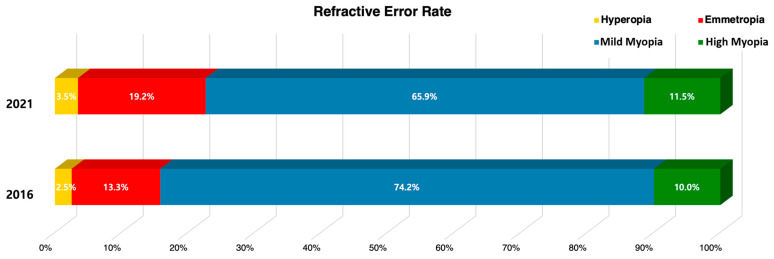
Changes in Refractive Error Prevalence Before and After the COVID-19 Pandemic.

The distribution of refractive error categories showed significant changes between the pre- and post-COVID-19 periods (adjusted F = 2.701, df1 = 2.869, df2 = 743.012, *p* = 0.047). Mean SE showed no statistically significant difference between 2016 (−2.77 ± 0.11 D, −10.63~+3.00 D) and 2021 (−2.63 ± 0.13 D, −14.00~+1.63, *p* = 0.443). The overall median SE indicated a slight reduction (–2.00 D to –1.75 D) In contrast, the median SE in the high myopia subgroup was –7.06 D in 2016 and –7.75 D in 2021 reflecting severity progression (Figure 2).

### 3.2. Participant Characteristics by Refractive Error Groups

Demographic and anthropometric characteristics varied significantly across refractive error groups (Table 1). Age, height, and weight showed significant differences across refractive error groups at both time points (*p* < 0.001). Participants with high myopia were consistently older and had greater height and weight compared to those with hyperopia and emmetropia at both survey periods.

Residential area was significantly associated with refractive error in 2016 (*p* = 0.049) but not in 2021 (*p* = 0.198), suggesting that regional differences in living or educational environments may have diminished after COVID-19. Sex was not significantly associated with refractive error groups in either 2016 (*p* = 0.974) or 2021 (*p* = 0.612). BMI categories showed significant associations with refractive error groups in 2016(*p* = 0.009) but lost significance in 2021(*p* = 0.125), indicating that the impact of BMI on refractive errors may have diminished during the pandemic period.

Sleep duration showed no significant differences among refractive error groups in 2016 (*p* = 0.261) but became significantly associated in 2021 (*p* = 0.047), with participants with high myopia having shorter sleep duration compared to those with hyperopia and emmetropia. Near work time was not significantly associated with refractive error groups in 2016 when assessed as combined reading and digital device time (*p* = 0.234).

In 2021, when assessed separately, only digital device usage time showed a significant association (*p* = 0.050), while paper-based reading time did not (*p* = 0.785).

### 3.3. Factors Associated with Refractive Errors

Univariate regression analysis revealed several factors significantly associated with refractive errors (Table 2). Age was the strongest predictor of refractive error severity at both time points (2016: *p* < 0.001; 2021: *p* < 0.001), with older age associated with more myopic refractive errors. The magnitude of this association increased from −0.193 D per year in 2016 to −0.324 D per year in 2021, indicating accelerated myopia progression with age during the post-COVID-19 period. Females had a significantly more myopic SE than males (−0.621 D, *p* = 0.019). Sleep duration was significantly protective against myopia at both time points (2016: *p* = 0.011; 2021: *p* = 0.011), with longer sleep duration associated with less myopic refractive errors. The protective effect increased from +0.262 D per hour in 2016 to +0.331 D per hour in 2021. Sex and residential area showed statistically significant differences only in 2021 (sex: *p* = 0.019; residential area: *p* = 0.001). BMI categories showed different patterns between the two periods. In 2016, obesity was associated with more myopic refractive errors (*p* = 0.022), while in 2021, underweight participants showed significantly more myopic refractive errors compared to normal weight participants (*p* = 0.015). Daily sedentary time and near work time were significant predictors in 2016 (sedentary time: *p* = 0.020; near work: *p* < 0.005) but lost significance in 2021. However, in 2021, digital device usage time for 4 h or more became significantly associated with more myopic refractive errors (*p* = 0.038).

Multivariate regression analysis showed different patterns between the two time periods (Table 3). Among the variables analyzed, near work time showed significant associations with refractive error when compared with the reference group of ≤1 h/day. In 2016, myopic shifts were significant only in the 3 h (B = –0.853, *p* = 0.040) and ≥4 h groups (B = –0.757, *p* = 0.045). In 2021, however, significant associations were already observed from the 1–2 h group (B = –1.612, *p* = 0.022) and persisted in the 3 h (B = –1.311, *p* = 0.042) and ≥4 h groups (B = –1.167, *p* = 0.014), with overall effects being stronger in the negative direction after the COVID-19 pandemic.

## 4. Discussion

This cross-sectional study analyzed changes in risk factors for refractive errors among Korean adolescents before and after the COVID-19 pandemic using data from the Korea National Health and Nutrition Examination Survey (KNHANES). After the pandemic, the overall prevalence of myopia decreased, whereas the proportion of high myopia increased. Although the mean spherical equivalent (SE) showed no significant difference, the median SE in the high myopia group shifted further toward the myopic direction. Even after adjusting for sex and residential area in the multivariate regression analysis, the association between age and refractive error became stronger after the COVID-19 pandemic (B = –0.185 → –0.312 D/year).

The most notable finding of this study was the post-pandemic increase in high myopia prevalence which aligns with several international studies [18,19]. Recent meta-analyses have reported that myopia progression in children and adolescents was significantly accelerated during the pandemic compared to pre-pandemic periods [13,14]. A population-based study from Hong Kong reported that myopia incidence in 6-year-old children increased 1.4–3 times during the pandemic compared to the previous 5-year average [20]. Additionally, research from China reported accelerated myopia progression in children aged 7–12 years during home quarantine [21]. European research reported no significant change in overall myopia prevalence among Dutch adolescents during the pandemic, but axial growth was accelerated, suggesting regional differences exist [15]. In contrast, our study found that overall myopia prevalence (mild and high myopia combined) decreased from 84.2% in 2016 to 77.4% in 2021 (*p* = 0.047), while the prevalence of high myopia increased. This indicates that although the total prevalence of myopia declined, the relative burden of high myopia among affected adolescents became greater, highlighting the increasing significance of severe myopia within the adolescent population. Furthermore, although the mean SE showed no statistically significant difference between survey years, the median SE of the high myopia subgroup indicated further progression, reflecting that mean values are influenced by the entire distribution, whereas subgroup medians better capture severity shifts. These changes may be partly due to the cross-sectional design of KNHANES and the use of non-cycloplegic refraction in adolescents, which could have influenced prevalence estimates. Nevertheless, KNHANES is a large-scale, nationally representative survey where cycloplegic refraction is not feasible. The consistent use of the same non-cycloplegic method in both years supports valid temporal and population-level comparisons, even if absolute diagnostic accuracy is limited.

Sex and residential area emerged as significant factors only after the COVID-19 pandemic, with female and urban adolescents showing a more pronounced tendency toward myopia. recent meta-analyses have increasingly reported higher myopia prevalence in females [10]. Female students showed higher myopia prevalence and more myopic SE [22], possibly linked to estrogen effects, with increased early puberty during the pandemic contributing [23]. In the univariate regression analysis, residential area was significant before the pandemic, but lost its significance after the pandemic. In the multivariate regression analysis, urban residents exhibited a greater tendency toward myopic refractive error than rural residents after the pandemic. As previous studies have indicated that access to green space may mitigate myopia by facilitating distance viewing during breaks from near work, the greater post-pandemic impact observed in urban areas may reflect limited opportunities for such visual breaks despite nationwide activity restrictions [24].

Regarding BMI, while obesity was associated with myopia before the pandemic, The 2016 pre-pandemic findings of our study may be partly consistent with the complex relationship between BMI and myopia reported in Korean children [16]. Interestingly, although the prevalence of obesity increased after the pandemic (12.8% to 20.1%, *p* = 0.029), more pronounced myopic progression was observed in the underweight group, whereas the obese group was not statistically significant in the multivariate regression. This shift may reflect multiple factors, including pandemic-related lifestyle and health changes (e.g., altered diet, reduced outdoor activity, and increased sedentary behavior), as well as methodological considerations such as non-cycloplegic refraction [20,25,26]. Although KNHANES employs probability-based sampling to ensure representativeness, some degree of sampling variation may also have contributed. Together, these factors highlight the need for cautious interpretation of this finding. Importantly, a nationwide Israeli study of 1.3 million adolescents demonstrated that both underweight and obese groups had a higher risk of myopia compared to those with normal BMI [27], suggesting that the association between BMI and myopia is nonlinear and context-dependent. Collectively, these findings indicate that this nonlinear pattern may have manifested differently before and after the pandemic. However, given the cross-sectional design, causal inferences cannot be established, and further longitudinal studies with detailed nutritional and lifestyle assessments are needed to clarify these associations [25,28].

It is important to note that our regression models explain relatively modest proportions of variance in refractive error (R^2^ = 7.9% in 2016; 13.4% in 2021), indicating that the included variables account for only a portion of the factors influencing refractive development. Appendix A provides additional analyses of sequential regression models in which sociodemographic variables identified in this study (BMI, near work time, sedentary behavior, and sleep duration) were incrementally included. Despite this stepwise inclusion, the explanatory power of the models increased only modestly (e.g., from 5.0% to 7.9% in 2016; from 12.2% to 13.4% in 2021), indicating that these variables account for only a limited proportion of the variance in refractive error. This suggests that other unmeasured genetic, environmental, or behavioral factors may play important roles in myopia development, and our findings should therefore be interpreted with appropriate caution regarding the strength of associations identified.

Additionally, the significant association when digital device usage exceeded 4 h aligns with the rapid risk increase in the 1–4 h interval presented in existing research [10]. Recent studies have reported that smartphone and tablet use may show different risk patterns compared to traditional near work [29], which is consistent with our finding that only digital device usage became a significant variable after the pandemic. Moreover, considering the protective effects of outdoor activities reported in systematic reviews [11,12], the restriction of outdoor activities during the pandemic may have contributed to these changes. Pandemic-related lifestyle changes are known to have affected various behavioral and growth indicators in adolescents [30]. In our study, the post-pandemic pattern of refractive error according to BMI differed from that reported in previous research [17], suggesting that such changes warrant consideration of nutritional assessments in conjunction with lifestyle factors when evaluating myopia risk. However, the assessment of near work differed between 2016 (a combined question on reading and computer use) and 2021 (separate questions on paper-based reading and digital device use), which limits direct comparability between the two periods. Although our approach of recombining the 2021 data for analysis was pragmatic, it may not have fully replicated the earlier measure. Therefore, although our analysis showed a stronger association between digital device use and myopia after the pandemic, this finding should be interpreted with caution in light of the methodological limitations of the assessment.

While sleep duration showed a protective effect in univariate analysis with the effect appearing stronger post-pandemic (+0.262 D in 2016; +0.331 D in 2021), this association did not remain statistically significant after adjusting for other variables in the multivariate model. This suggests that the apparent protective effect of sleep may be mediated through or confounded by other factors such as age, lifestyle patterns, or overall health behaviors, highlighting the complex interrelationships among variables affecting refractive development. Previous studies similarly found no direct effect of sleep duration on myopia, though related factors were associated [31]. Our findings also emphasize the need to assess the impact of sleep on SE while controlling for various confounding variables. Likewise, stress perception and sedentary time were not statistically significant after adjusting for age and were excluded from the final analysis, suggesting that the effect of age on refractive error became more pronounced after the pandemic.

The main strength of this study is the direct comparative analysis of changes before and after the COVID-19 pandemic using large-scale data with national representativeness. Additionally, we obtained results representative of all Korean adolescents through statistical analysis reflecting complex sampling design and ensured research quality through systematic reporting following STROBE guidelines. The application of complex sample statistical methods appropriately addressed the multi-stage stratified sampling design, enhancing the validity and generalizability of our population estimates. While existing studies were mainly conducted at single institutions or regional levels, this study enhanced generalizability through a nationwide sample. However, this study has several limitations. The cross-sectional design comparing two independent cohorts from 2016 and 2021 precludes establishing causal relationships and cannot determine whether observed differences reflect pandemic-related lifestyle changes or simply differences between the sampled populations. Refractive errors were measured without cycloplegia, which may have led to an overestimation of myopia and an underestimation of hyperopia, particularly in adolescents. This limitation should be considered when interpreting the results, especially the discrepancy between the decreased overall myopia prevalence and increased high myopia. However, in large-scale nationwide surveys such as KNHANES, conducting non-cycloplegic refraction is more appropriate, and the consistent application of the same non-cycloplegic method in both survey periods provides meaningful value to the study [26]. And self-reported variables may be subject to measurement errors or recall bias. In addition, differences in the assessment methods for near work between 2016 (combined tasks) and 2021 (separated screen and reading tasks) limit direct comparability between the two periods.

## 5. Conclusions

This study suggests that the COVID-19 pandemic was accompanied by shifts in refractive error patterns and associated risk factors among Korean adolescents. After the pandemic, high myopia prevalence increased, and the association between age and refractive error strengthened, accelerating myopia progression. In addition, digital device usage showed stronger associations with myopia than traditional paper-based near work. These findings highlight potential changes in the influence of sociodemographic and behavioral factors following the pandemic; however, given the cross-sectional design, causal interpretations remain limited, and longitudinal studies are needed to confirm these associations.

## Figures and Tables

**Table 1:** Characteristics of adolescents according to refractive errors before and after the COVID-19 pandemic (2016 and 2021).

Variables		Pre–COVID-19 (2016)						Post–COVID-19 (2021)					
		Hyperopia	Emmetropia	Mild Myopia	High Myopia	Total ^e^	*p*	Hyperopia	Emmetropia	Mild Myopia	High Myopia	Total ^e^	*p*
Refractive error	N	20	100	505	66			19	93	329	49		
	mean ± SD	1.24 ± 0.19	−0.04 ± 0.03	−2.78 ± 0.08	−7.30 ± 0.17			0.74 ± 0.08	−0.05 ± 0.03	−2.64 ± 0.10	−7.93 ± 0.31		
	range (D)	+0.50~+3.00	−0.38~+0.38	−5.88~−0.50	−10.63~−6.00			+0.50~+1.63	−0.38~+0.38	−5.88~−0.50	−14.00~−6.00		
Gender, n (%) ^a^	Male	9 (2.52)	54 (13.23)	261 (74.83)	34 (9.42)	358 (52.6)	0.974	12 (3.97)	53 (20.92)	172 (65.23)	21 (9.88)	258 (51.0)	0.612
	Female	11 (2.51)	46 (13.37)	244 (73.54)	32 (10.58)	333 (47.4)		7 (2.94)	40 (17.37)	157 (66.59)	28 (13.10)	232 (49.0)	
Area of residence, n (%) ^a^	Urban	14 (1.97)	82 (12.56)	437 (75.4%)	56 (9.99)	589 (87.4)	0.049	13 (3.06)	71 (17.96)	269 (66.79)	42 (12.18)	395 (85.7)	0.198
	Rural	6 (6.28)	18 (18.42)	68 (65.45)	10 (9.86)	102 (12.6)		6 (5.85)	22 (26.46)	60 (60.56)	7 (7.13)	95 (14.3)	
Age ^b^		12.91 ± 0.47	13.56 ± 0.38	14.29 ± 0.11	15.37 ± 0.33		0.000	12.29 ± 0.59	12.93 ± 0.30	13.87 ± 0.16	15.46 ± 0.33		0.000
Height ^b^		159.49 ± 3.17	157.22 ± 1.35	161.73 ± 0.49	166.70 ± 1.30		0.000	152.10 ± 2.76	157.52 ± 1.50	161.89 ± 0.78	165.04 ± 1.14		0.000
Weight ^b^		51.01 ± 2.80	51.23 ± 1.87	54.71 ± 0.72	63.57 ± 2.14		0.000	47.01 ± 3.15	53.63 ± 2.18	57.80 ± 1.40	60.05 ± 2.05		0.001
BMI ^b^		19.83 ± 0.74	20.38 ± 0.53	20.65 ± 0.22	22.74 ± 0.64		0.009	19.91 ± 0.77	21.18 ± 0.64	21.73 ± 0.38	21.97 ± 0.69		0.125
BMI Group, n (%) ^a^	low	2 (2.90)	9 (16.64)	48 (71.28)	6 (9.18)	65 (10.0)	0.232	-	5 (17.59)	18 (64.86)	6 (17.55)	29 (7.1)	0.805
	normal	16 (3.01)	70 (12.78)	356 (76.31)	39 (7.91)	481 (68.4)		13 (4.29)	59 (19.37)	210 (65.15)	29 (11.19)	311 (63.5)	
	overweight	-	8 (15.50)	45 (71.12)	8 (13.38)	61 (8.8)		1 (1.44)	12 (21.07)	40 (72.00)	3 (5.49)	56 (9.3)	
	obesity	2 (1.33)	13 (11.94)	56 (67.51)	13 (19.22)	84 (12.8)		5 (3.02)	17 (18.41)	60 (65.57)	11 (13.00)	93 (20.1)	
Home income, (%) ^a^	Q1	4 (3.66)	4 (4.15)	57 (82.82)	7 (9.37)	72 (11.8)	0.051	1 (0.92)	4 (7.76)	25 (82.62)	3 (8.70)	33 (6.2)	0.328
	Q2	3 (1.39)	37 (21.25)	118 (71.62)	7 (5.73)	165 (24.5)		4 (2.62)	23 (20.02)	84 (65.88)	11 (11.48)	122 (25.4)	
	Q3	8 (3.47)	27 (11.88)	162 (74.14)	23 (10.52)	220 (31.7)		11 (6.20)	38 (21.17)	118 (62.63)	16 (10.00)	183 (37.1)	
	Q4	5 (2.03)	32 (12.06)	167 (73.16)	28 (12.75)	232 (32.0)		3 (1.43)	28 (18.51)	101 (66.29)	19 (13.76)	151 (31.3)	
Sleep duration (hour) ^b,c,d^		7.87 ± 0.37	7.60 ± 0.15	7.62 ± 0.07	7.26 ± 0.19		0.261	8.41 ± 0.42	7.59 ± 0.24	7.49 ± 0.09	7.31 ± 0.20		0.047
Rate of Perceived Stress, n(%) ^a,c,d^	Yes	8 (2.03)	44 (10.46)	278 (74.70)	48 (12.81)	378 (72.9)	0.557	9 (2.90)	48 (15.84)	189 (66.71)	34 (14.54)	280 (77.0)	0.895
	No	2 (1.33)	20 (14.31)	107 (74.16)	15 (10.21)	144 (27.1)		2 (1.65)	13 (15.86)	58 (69.23)	11 (13.26)	84 (23.0)	
Average Daily Sedentary Time (hour) ^b,c,d^		9.77 ± 1.29	10.51 ± 0.36	10.89 ± 0.18	11.38 ± 0.37		0.290	10.53 ± 1.27	10.98 ± 0.35	11.38 ± 0.16	11.64 ± 0.35		0.499
Near Work (hour), n (%) ^a,†^	≤1	1 (1.90)	12 (26.46)	28 (68.44)	1 (3.20)	42 (5.6)	0.234	6 (6.08)	19 (20.68)	65 (63.84)	9 (9.39)	99 (19.8)	0.785
	1–2 h	6 (2.47)	32 (15.86)	125 (74.86)	13 (6.81)	176 (24.9)		2 (1.98)	16 (22.40)	45 (61.92)	9 (13.70)	72 (16.7)	
	3 h	4 (2.13)	16 (11.50)	121 (74.72)	17 (11.64)	158 (22.8)		3 (4.58)	7 (13.19)	40 (66.25)	7 (15.98)	57 (13.5)	
	≥4	9 (2.80)	40 (11.22)	231 (74.33)	35 (11.65)	315 (46.7)		8 (2.62)	51 (19.12)	179 (67.95)	24 (10.31)	262 (50.0)	
Near Work (hour), n (%) ^a,‡^	≤1							2 (13.41)	6 (35.02)	13 (51.57)	-	21 (3.6)	0.050
	1–2 h							2 (2.13)	18 (21.81)	43 (70.46)	4 (5.60)	67 (13.9)	
	3 h							3 (1.63)	21 (22.71)	74 (68.98)	7 (6.68)	105 (19.9)	
	≥4							12 (3.77)	48 (16.56)	199 (64.73)	38 (14.94)	297 (62.6)	

Continuous variables are presented as weighted means ± standard errors across all participants. ^a^ *p*-values were calculated using the Rao-Scott Chi-square test. ^b^ *p*-values were calculated using the Complex Samples General Linear Model. ^c^ Data from the 2016 survey were obtained from adolescents aged 12 years and older. The number of participants included in the analysis after excluding user-defined missing values was as follows: sleep duration (*n* = 521), perceived stress (*n* = 522), and average daily sedentary time (*n* = 519). ^d^ Data for 2021 were collected from adolescents aged 12 years and older. After excluding user-defined missing values, 364 participants were included in the analysis of both sleep duration, average daily sedentary time and perceived stress. ^e^ Total columns presents the number of participants and weighted percentages for categorical variables. † In 2016, near work duration included both paper- and screen-based activities; in 2021, only paper-based near work was surveyed. ‡ Variables surveyed only in 2021 (display near work time).

**Table 2:** Univariate regression analysis of factors associated with refractive errors before and after the COVID-19 pandemic (2016 and 2021).

Variables (Reference)		Pre-COVID 19 (2016)			Post-COVID 19 (2021)		
		B	95% CI	*p*-Value	B	95% CI	*p*-Value
Sex (Female)		0.190	−0.168~0.548	0.296	0.621	0.105~1.137	0.019
Town (rural)		−0.441	−1.233~0.351	0.274	−0.865	−1.365~−0.365	0.001
Age		−0.193	−0.275~−0.111	0.000	−0.324	−0.429~−0.218	0.000
BMI Group (normal)	Low	0.648	−0.102~1.399	0.090	−1.327	−2.394~−0.260	0.015
	Overweight	−0.340	−1.087~0.408	0.372	0.252	−0.669~1.173	0.589
	Obesity	−0.741	−1.373~−0.109	0.022	−0.344	−1.118~0.431	0.381
Home income (Q1, lowest)	Q2	0.455	−0.194~1.104	0.169	0.537	−0.411~1.485	0.270
	Q3	0.053	−0.627~0.733	0.878	0.882	0.064~1.699	0.030
	Q4	−0.174	−0.810~0.462	0.591	0.057	−0.841~0.955	0.900
Sleep duration		0.262	0.062~0.462	0.011	0.331	0.077~0.584	0.011
Rate of Perceived Stress (No)		0.130	−0.396~0.657	0.625	−0.104	−0.855~0.647	0.785
Average Daily Sedentary Time		−0.105	−0.194~−0.017	0.020	−0.063	−0.164~0.039	0.225
Near Work 1 (≤1 h/day) ^†^	1–2 h	−0.845	−1.659~−0.031	0.042	0.029	−0.898~0.956	0.951
	3 h	−1.150	−2.035~−0.265	0.011	−0.063	−0.990~0.864	0.894
	4≤	−1.171	−1.951~−0.390	0.003	−0.080	−0.830~0.671	0.835
Near Work 2 (≤1 h/day) ^‡^	1–2 h				−0.853	−2.359~0.654	0.266
	3 h				−0.913	−2.380~0.555	0.222
	4≤				−1.542	−2.998~−0.086	0.038

^†^ In 2016, near work duration included both paper- and screen-based activities; in 2021, only paper-based near work was surveyed./^‡^ Variables surveyed only in 2021 (display near work time). Note: Regression coefficients represent the change in spherical equivalent (in diopters) associated with each unit change in the predictor variable, with negative values indicating more myopic refractive errors.

**Table 3:** Multivariate regression analysis of factors associated with refractive errors before and after the COVID-19 pandemic (2016 and 2021).

Variables (Reference)		Pre–COVID-19 (2016)			Post–COVID-19 (2021)		
		B	95% CI	*p*-Value	B	95% CI	*p*-Value
Adjusted Mean		0.915	−0.517~2.347	0.208	3.866	2.247~5.485	0.000
Sex (Female)		0.207	−0.143~0.557	0.244	−0.544	−1.039~−0.050	0.031
Town (rural)		−0.434	−1.156~0.288	0.237	−0.685	−1.229~−0.142	0.014
Age		−0.185	−0.268~−0.102	0.000	−0.312	−0.422~−0.203	0.000
BMI Group (normal)	Low	0.720	0.027~1.412	0.042	−0.948	−1.809~−0.088	0.031
	Overweight	−0.355	−1.131~0.421	0.367	−0.017	−0.954~0.920	0.971
	Obesity	−0.650	−1.256~−0.044	0.036	−0.407	−1.169~0.355	0.292
Near Work time ^†^ (≤1 h/day)	1–2 h	−0.716	−1.436~0.032	0.060	−1.612	−2.986~−0.238	0.022
	3 h	−0.853	−1.666~−0.041	0.040	−1.311	−2.571~−0.051	0.042
	4≤	−0.757	−1.499~−0.016	0.045	−1.167	−2.094~−0.241	0.014
		F(P) = 5.212(0.000), R^2^ = 7.9%			F(P) = 9.134(0.000), R^2^ = 13.4%		

^†^ Near work time was assessed as combined screen and paper-based tasks in 2016, and separately in 2021. For comparability, 2021 data were recalculated and combined to match the 2016 definition.

## Data Availability

Data were obtained from the following website: https://knhanes.kdca.go.kr/knhanes/eng/main.do (accessed on 1 August 2025).

## Appendix A

**Table A1:** R^2^ of sequential regression models for refractive error before and after the COVID-19 pandemic (2016 vs. 2021).

No.	Variables	Pre-COVID-19 (2016)	Post-COVID-19 (2021)
Model 1	Sex, town, age	5.0%	12.2%
Model 2	model 1+ BMI	7.3%	13.2%
Model 3	model 1+ near work time	5.8%	12.4%
Model 4	model 2 + near work time	7.9%	13.4%
Model 5	model 4 + Average Daily Sedentary Time	6.4%	11.5%
Model 6	model 5 + sleep duration	6.6%	11.6%
